# The advantage of objects over images in discrimination and reversal learning by kea, *Nestor notabilis*

**DOI:** 10.1016/j.anbehav.2014.12.022

**Published:** 2015-03

**Authors:** Mark O'Hara, Ludwig Huber, Gyula Kopanny Gajdon

**Affiliations:** a Messerli Research Institute, University of Veterinary Medicine Vienna, Medical University Vienna, University of Vienna, Vienna, Austria; b Department of Cognitive Biology, University of Vienna, Vienna, Austria

**Keywords:** behavioural flexibility, discrimination learning, kea, *Nestor notabilis*, reversal learning, touchscreen

## Abstract

Studies investigating the same paradigm but employing different methods are often directly compared in the literature. One such paradigm used to assess behavioural flexibility in animals is reversal learning. Commonly, these studies require individuals to learn the reward contingency of either solid objects presented on the ground or images presented on a touchscreen. Once learned, these contingencies are swapped. Researchers often refer to trials required to reach learning criteria from different studies, to compare the flexibility of different species, but rarely take methodological differences into account. A direct evaluation of the validity of such comparisons is lacking. To address this latent question, we confronted kea, an alpine parrot species of New Zealand and known for its behavioural flexibility, with a standard reversal learning paradigm on the touchscreen and a standard reversal learning paradigm with solid objects. The kea required significantly more trials to reach criterion in the acquisition and the reversal on the touchscreen. Also, the absolute increase in the number of trials required for the reversal was significantly greater on the touchscreen. This indicates that it is not valid to compare learning speed across studies that do not correspond in the addressed methodology. Taking into account the kea's ecology and explorative nature we discuss stimulus abstraction (limited depth cues and tactile stimulus feedback) and the spatial relation between reward and stimulus on the touchscreen as possible causes for decreased inhibition in this condition. Contrary to the absolute increase in number of trials required for the reversal, the increase in relation to the acquisition was greater with solid objects. This highlights the need for further research on the mechanisms involved causing methodology-dependent differences, some of which we discuss, in order to increase the validity of interpretations across studies and in respect to the subject's ecology.

## Highlights

• We provide a first direct comparison of two standard reversal learning approaches.
• Discrimination and reversal of solid objects seems easier than on the touchscreen.
• This highlights issues when comparing the same paradigm using different approaches.

Research on animal cognition is mainly divided into social cognition and individual problem solving. Problem solving without investigating tool use is largely concerned with tasks involving decision making. Such choice tasks may focus on behaviours directed towards different solid objects (like cups, strings, etc.) or make use of abstract stimuli in certain domains of perception, such as sounds, olfaction or, most commonly, two-dimensional visual stimuli. To present such visual stimuli to birds, special disks with sensors, so-called ‘pecking keys’ (e.g. Zentall & Hogan, 1975) can be used. Such pecking keys are more and more being replaced by the use of touchscreens (e.g. Cook, 1992; Cook, Geller, Zhang, & Gowda, 2004; Fagot & Paleressompoulle, 2009; Gibson, Wasserman, Frei, & Miller, 2004; see Steurer, Aust, & Huber, 2012 for a review) to be able to present multiple stimuli in different locations. The touchscreen approach greatly benefits data collection efficiency, reduces or eliminates possible experimenter bias and may aid interspecies comparability of tasks (e.g. Bond, Wei, & Kamil, 2010; Markham, Butt, & Dougher, 1996; McGregor & Healy, 1999; Steurer et al., 2012; Sutcliffe & Hutcheson, 2012; Vonk, 2013).

Solid objects, in contrast, offer additional qualities like olfaction and haptic experience that can be utilized in addition to vision to discriminate objects (e.g. Fagot, Drea, & Wallen, 1991; Uchida & Mainen, 2003). But in regard to the visual domain there remains a qualitative difference between objects and images. Solid objects offer more salient depth cues, which may be recognized through binocular disparity used in stereoscopic vision, motion parallax or accommodation and convergence of the eyes (D'Eath, 1998; Friedman, Spetch, & Ferrey, 2005; McFadden, 1993; Watanabe, 1997). When tested with novel views of objects and their photographs, pigeons, *Columba livia*, performed faster and more accurately with objects (Friedman et al., 2005) and the authors suggested that the processes leading to the representations in the two domains might differ. Based on these and other results, Spetch and Friedman (2006a) concluded that real objects would be easier for pigeons to discriminate. Interestingly, Stephan, Steurer, and Aust (2014) also recently found, in a carefully controlled study, that pigeons were better in discriminating real objects than their holographic representations. This is intriguing, since depth cues should not differ between objects and their holographs.

Despite these obvious differences in stimulus qualities, so far, to our knowledge, research focusing on the comparability of the same task employing different methods is lacking. Nevertheless, conclusions have frequently been derived from the comparison of studies involving different methodologies in the literature (e.g. Tebbich, Sterelny, & Teschke, 2010). To date, the only indirect information regarding this issue comes from comparisons of objects with two-dimensional representations, which are limited to studies concerned with picture–object equivalence (see Fagot, 2000; and for a thorough review: Bovet & Vauclair, 2000; Weisman & Spetch, 2010). Furthermore, these experiments have been conducted mainly on pigeons (Aust & Huber, 2006; Cabe, 1976; Delius, Emmerton, Hörster, Jäger, & Ostheim, 1999; Spetch & Friedman, 2006b; Stephan, Wilkinson, & Huber, 2013; Watanabe, 1997, 2000) or nonhuman primates (e.g. Fagot, Martin-Malivel, & Dépy, 2000; Zimmerman & Hochberg, 1970, 1971). But compared to pigeons whose default natural response is to peck at objects, more manipulative species might be more influenced by tactile feedback. Rats, *Rattus norvegicus*, for example, perform better in an olfactory set-up than when they have to learn visually (Eichenbaum, Fagan, & Cohen, 1986), thus highlighting the importance of species-specific expertise in certain modalities. Tactile feedback has been shown to play an important role in common object recognition, at least in humans (Klatzky, Lederman, & Metzger, 1985; Lederman & Klatzky, 1987). This haptic element might benefit the association formation and discrimination learning of three-dimensional objects over images and may accommodate especially extractive foragers such as the kea.

The kea, a parrot species endemic to New Zealand, lives in mountainous to alpine environments that are subject to major seasonal changes. Therefore kea have specialized in applying their skills to new circumstances and are considered a hallmark example of an ‘open program’ species (Diamond & Bond, 1999). This environment has rendered kea generalists that feed on many different plant species and also dig with their long beaks in the ground to access roots (Brejaart, 1988), and especially adults have been observed excavating food sources (Diamond & Bond, 1991), which qualifies them to be considered extractive foragers. In contrast to pigeons, the highly manipulative (or rather ‘rostripulative’) kea have developed different strategies of interacting with stimuli on a touchscreen (M. O'Hara, personal observation). Apart from having the basic drive to deconstruct the frame of the screen, kea select stimuli by touching them with the base or tip of the beak, with the tongue or even with the feet. Gajdon, Amann, and Huber (2011) recently also demonstrated the kea's behavioural flexibility in a reversal learning task involving tools by showing that these parrots rely on social information at first, but abandon it in favour of overt exploration. Different levels of neophilia and hence readiness to explore novel options have been suggested to be underlying factors accounting for behavioural flexibility in a problem-solving experiment comparing kea and New Caledonian crows, *Corvus moneduloides* (Auersperg, von Bayern, Gajdon, Huber, & Kacelnik, 2011).

To be able to behave in such a flexible and explorative way, a corresponding cognitive set-up is necessary, which can be experimentally investigated through reversal learning (Berg, 1948; Bond, Kamil, & Balda, 2007). Numerous studies have been published using reversal learning to investigate flexibility in a wide range of different animal species (e.g. rhesus monkeys, *Macaca mulatta*: Herndon, Moss, Rosene, & Killiany, 1997; Göttingen minipig, *Sus scrofa*: Moustgaard, Arnfred, Lind, Hansen, & Hemmingsen, 2004; North American corvids (*Gymnorhinus cyanocephalus, Nucifraga columbiana, Aphelocoma californica*): Bond et al., 2007; rats: Floresco, Block, & Tse, 2008; capuchin monkeys, *Cebus apella*: Beran et al., 2008; humans: Kloo, Perner, Kerschhuber, Dabernig, & Aichhorn, 2008; kea: Gajdon et al., 2011). Reversal learning is defined as the reversal of an original problem after reaching a learning criterion in the training phase (Sutherland & Mackintosh, 1971). Hence individuals are required to reverse their former associations by inhibiting responses towards formerly rewarded stimuli, shift their attention and form a new association with previously unrewarded stimuli (Lai, Moss, Killiany, Rosene, & Herndon, 1995). Brain lesion studies gave insight to the question which parts of the brain are involved in reversal learning (for a review see Watanabe, 2006, 2012). Macphail (1971, 1976) concluded that the hyperstriatal complex in pigeons is involved in reversal learning, by inhibiting responses, rather than shifting the attention. Similarly, Dias, Robbins, and Roberts (1996) concluded poor performance in reversal learning tasks resulted from a lack of inhibitory control mediated by the prefrontal cortex in marmosets, *Callithrix jacchus*. In more recent studies, Watanabe (2003) investigated what kind of cognitive mechanisms and neuronal basis might underlie cognitive flexibility in birds. His study indicated that lesions in the Wulst and hippocampus lead to impairments in the consolidation phase, whereas lesions in the basal ganglion reveal deficits in the consolidation, but even stronger so in the search phase of repeated acquisitions. Beran et al. (2008) concluded from the results of their carefully designed noninvasive study that reversal learning in capuchins (on the touchscreen) rather employs associative processes than rule-based mechanisms. Okada et al. (2014) have recently demonstrated that the selective eliminations of striatal cholinergic interneurons of the dorsomedial striatum of rats led to improvements in behavioural switching of spatial discrimination reversal and extinction learning, highlighting the inhibitory role of striatal cholinergic interneurons in behavioural flexibility.

But while some have investigated differences in spatial and pattern (images) or spatial and object reversals (Herndon et al., 1997; Lai et al., 1995) the performance in the same task using objects and images remains unstudied. Considering the lack of direct methodological comparisons the main aim of this study was to address the comparability of the same paradigm investigated with differing methodologies, namely presenting images on a touchscreen as compared to using solid objects. To achieve this goal and evaluate previous comparisons of studies employing the same paradigm but involving different procedures (e.g. Spetch, 1995; Tebbich et al., 2010) we chose two standard methods used to investigate flexibility in animals. The previously argued advantage of three-dimensional objects over images might be especially pronounced in more difficult tasks such as reversal learning. Taking into account the kea's manipulative nature and considering the additional haptic dimension of solid objects we would expect the kea to perform better in this more naturalistic set-up where they might profit from being extractive foragers.

Seasonal changes in the environment force the kea to explore many different food sources and flexibly adapt their foraging strategies. However, for extractive foragers, which put in more effort to reach certain roots, it seems adaptive to occasionally revisit a previously rewarded stimulus (e.g. plant) even if not being rewarded immediately (Winkler & Leisler, 1999). Inhibiting responses to stimuli that were associated with a reward at some point, thus fully reversing the discrimination, might be more challenging for these animals than for nonextractive foragers (Gajdon et al., 2011; Tebbich et al., 2010). Therefore we expected the kea to form associations quickly and readily shift their attention to the former unrewarded stimulus, but they might require a long time to inhibit previously rewarded responses and to complete the reversal task.

## Methods

### Ethical Note

All subjects that participated in our experiments are housed in accordance with the Austrian Federal Act on the Protection of Animals (Animal Protection Act—TSchG, BGBl. I Nr.118/2004). Furthermore, as the present study was strictly noninvasive and based on behavioural observations, all experiments were classified as nonanimal experiments in accordance with the Austrian Animal Experiments Act (§ 2, Federal Law Gazette No. 501/1989).

### Test Subjects

The experiments were carried out with 20 captive kea (see Table 1) at the Konrad Lorenz Institute for Comparative Ethology (KLIVV) in Vienna, Austria. They were housed together in a large, environmentally enriched group aviary (15 × 10 m and 4 m high) that could be divided into three equally sized experimental compartments. The birds received a diet of fruit, vegetable, protein and seed every day. Drinking water and bathing opportunities were available ad libitum. Eight birds had previously participated in discrimination experiments on the touchscreen (O'Hara, Gajdon, & Huber, 2012), but had not received any reversal learning tasks on it. The other 12 individuals were naïve to the touchscreen and therefore received pretraining (see Touchscreen training section). Apart from four individuals (Plume, Roku, Rosa and Willy), the kea had participated earlier in another reversal task involving solid objects conducted by Gajdon et al. (2011). To control for differing experience we included them as factors in our model.

We excluded four individuals from the experiments because of health problems (Mismo and Zappel), because they failed habituation attempts in the solid object condition (Roku) or because of technical difficulties (Rudy).

### General Procedure

In each condition (touchscreen or solid objects) the individuals had to complete two phases (the acquisition and the reversal phase). The learning criterion was set to 85% correct first choices in two consecutive sessions. Upon reaching this criterion the subjects were transferred into the next phase or condition, respectively. In the acquisition phase the kea had to discriminate between two stimuli by choosing the rewarded one (S+). In the following reversal phase they were required to choose the former unrewarded stimulus (S–), while the former rewarded stimulus became unrewarded.

Two different sets of stimuli were used, meaning that individuals were not confronted with the same stimuli in both conditions. Which stimulus set was used (as well as which condition was conducted first) had been pseudorandomly assigned to each individual, but counterbalanced between age and sex groups. One daily session consisted of 20 trials and a trial was repeated until the correct choice was made, but only counted as correct if the first choice was correct. A correct choice was rewarded with an eighth of a peanut seed in both conditions. Birds were tested individually, out of sight of their conspecifics.

The experiment was divided into two conditions: (1) the ‘touchscreen condition’, in which the kea had to touch visual stimuli presented on a computer screen in order to make a choice and (2) the ‘solid object condition’, in which they had to turn over the correct plastic cups to reach the reward hidden underneath.

### The Touchscreen Condition

#### Apparatus

**Figure 1:**
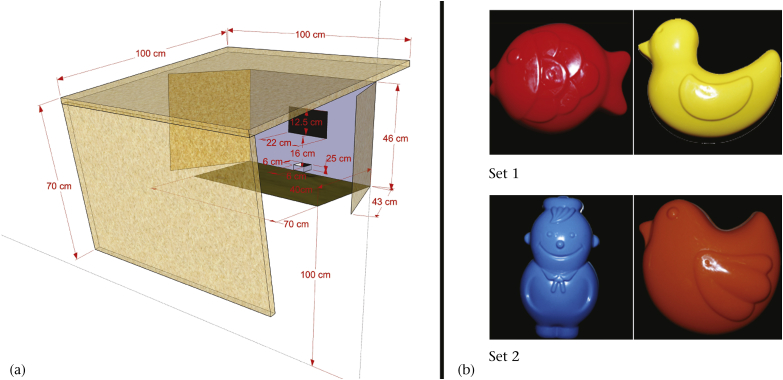
(a) The touchscreen apparatus and its dimensions: the black rectangle shows the position of the touchscreen, with the feeding tray positioned below; the inner sides of the side and hind flaps were painted black to reduce reflection of sunlight. (b) The two stimulus sets as images used on the touchscreen.

To interact with the touchscreen the subjects had to enter a cabin with flaps arranged in order to avoid reflections of the sun (Fig. 1a), located in the experimental compartment of the outdoor aviary, and stay on a platform located in front of the touchscreen (70 × 40 cm, 1 m above ground). From here they could access a 15-inch XGA colour TFT computer screen (Model G150XG01 produced by AU Optronics Corp., Taiwan), with a display area of 304 mm × 228 mm (381 mm diagonal) and a resolution of 1024 × 768 pixels. Attached to the frontal frame of the screen was a 15-inch IR touch frame (Model ‘CarrollTouch’ D87587-001, 15 in.) produced by Elo (Menlo Park, CA, U.S.A.) for detecting responses. The IR grid was located directly in front of the safety glass plate, which protects the LCD display from damage and dirt. The monitor and all described components were installed within a dust-proof, fan-less (passively cooled) anodized aluminium case (measuring 39 × 8 cm and 30 cm high). The screen was connected to a modified operant conditioning system described in detail by Steurer et al. (2012). The CPU (based on a Schneider A4F minicomputer (http://www.mappit.de) with Mini-ITX main board (VIA EPIA1 M10000, with 1 GHz CPU, 2 × USB, 1 × LAN 10/100 Mbit, sound and VGA on board), 512 MB DDR RAM, a 40 GB 2.5-inch hard disc) and feeding system, attached behind the touch-sensitive screen, were contained in a sealable plastic cube resting outside the aviary, secured to a metal frame embedded in the wire mesh of the aviary wall. The feeding system consisted of a motor, sensor and circular plastic disc with holes, which would rotate one reservoir further, thus releasing a reward into a small tray (60 × 60 mm and 30 mm deep) below the screen whenever a stimulus with positive contingency was touched. The opening for delivering the reward and tray were located centrally 160 mm below the lower edge of the screen.

We used CogLab light (version 1.4; see Steurer et al., 2012 for detailed description) to control the operation of the feeder and the touch frame, to present the stimuli and to record the responses (see below).

#### Stimuli

For touchscreen training (see below) bitmaps, 140 × 140 pixels in size (3.7 cm × 3.7 cm), consisting of a simple geometric figure (a circle, a triangle, etc.) were used.

For the acquisition and reversal of the actual task two sets of two images each were used. The images were photographs of the objects that were used in the solid object condition (see Fig. 1b). They had been edited by Photoshop Elements v.6.0 to remove the background. The trigger stimulus at the beginning of each trial consisted of a white cross 140 × 140 pixels in size.

#### Procedure

##### Touchscreen training

The training phase consisted of two sessions of 35 trials each. As a reward a maximum of 12 peanuts were given to each bird (whole seeds, half, quarter or eighth pieces of seeds were randomly distributed among trials). The intertrial interval (ITI), during which the screen went black, was set to 1 s. When a bird left the platform and did not return to the touchscreen within 10 min, the session was aborted and restarted from the same point the following day.

In the first eight trials the birds were presented with a single starting stimulus. These images were shown centrally on the screen in the first eight trials and also included additional stimulus/local enhancement by the experimenter (M.O.) moving a mouse cursor. After this initial phase, the image was presented at a randomized position and with the mouse cursor hidden for the rest of the session (27 trials). For the second habituation session these conditions remained unchanged, except that only a total of four peanut seeds were delivered during this session per bird (an eighth of a seed per trial).

##### Acquisition and reversal

After completing the touchscreen training each bird was confronted with a new set of stimuli (Fig. 1b). When the bird touched the centred trigger stimulus (which triggered the beginning of each trial from which we calculated exact response latencies) the actual stimuli were presented. These stimuli were located on the medium horizontal axis of the screen, one-third of the screen's length from the left frame, and the other one two-thirds of the screen's length from the left frame, leaving approximately 10 cm of space in between. The side on which each stimulus was shown was semirandomly assigned. To prevent the subjects from developing side preferences, correction trials (CT) were provided after each incorrect choice until the correct stimulus was pecked. The correction intertrial interval was set to 2 s. A peck on the positive stimulus (S+) was rewarded by the delivery of an eighth piece of a peanut into the touchscreen reward tray. Additionally, every choice (i.e. the breaking of the IR beams) was also paired with an acoustic feedback. For this purpose, we recorded the sound of knocking by hand on the solid object that was depicted (see below), which sounded slightly different for each stimulus depending on the shape.

After the subject reached the learning criterion of 85% correct first choices in each of two consecutive sessions, the reward contingencies were reversed (a former S+ became an S− and vice versa). The acoustic feedback for each stimulus choice remained the same.

##### Data recording

The CogLab program (see above) automatically logged date and time during the experiments and counted the correct first choices per session and the correction trials (the number of errors per trial). Additionally, the location of the stimuli and the frequency of touching the screen beside an image (pecks on screen) were recorded.

### The Solid Object Condition

#### Stimuli

Two coloured plastic cups were placed in the sand on the floor of the experimental compartment, centrally within a circle trenched in the sand (100 cm in diameter). They were placed 40 cm apart on an imaginary line at a right angle to the bird's normal approach so that we could clearly determine which stimulus was chosen. The plastic cups were an orange chick, a blue man, a red fish and a yellow duck-like shape and were commercial PVC forms for toddlers (12.2 cm in width and 4.1 cm in height). The ratio of spacing in respect to stimulus size (0.31) therefore remained similar to the ratio between size and distance of the stimuli on the touchscreen (0.37).

#### Procedure

##### Cup habituation

Because of the explorative nature of the kea, they readily investigated the cups upon first encounter and therefore nearly no habituation was needed in this condition. If there was no response in the very first trial, a piece of peanut was placed in the centre of the circle between the cups. This provided enough motivation for them to become interested in the stimuli.

##### Acquisition and reversal

The reward (an eighth of a peanut) was placed beneath the S+ and in order to retrieve it a subject had to turn over the correct object. Lifting the unrewarded stimulus was considered an error, but the trial continued, similarly to the correction trials of the touchscreen task, until the correct stimulus was turned and the reward was retrieved.

After each trial the subject was removed from the experimental compartment for approximately 30 s. During this time the stimuli were rebaited and rearranged for the next trial out of the subject's sight, behind an opaque barrier. The position (right or left) of the stimuli was again pseudorandomized, with the S+ being placed on the same side in no more than three consecutive trials.

##### Data recording

Each trial was videotaped and analysed in cases of uncertainty of mistakes or latencies. The date, the side of the positive stimulus and the number of errors per session were noted. By using a commercial stopwatch we timed the subjects entering the experimental compartment, entering the circle, the first choice and any further choices (if any), as well as the total trial time. Time to decision was defined as the time between entering the circle and the first lift of any cup. During each trial the experimenter (M.O.) remained in the compartment adjacent to the experimental compartment.

### Data Analysis

As a measure of performance in the different phases (acquisition or reversal) and conditions (touchscreen or solid objects) the number of trials until the last incorrect choice before reaching criterion (trials to criterion; TTC) was investigated. To meet the assumptions of normality for modelling, data were log(*X*+1) transformed.

To explore the effects of previous experience with the touchscreen (O'Hara et al., 2012), influence of previous reversal learning tasks (Gajdon et al., 2011) with solid objects, sex, age, stimulus set (with which set individuals started), group (in which condition individuals started), phase and condition on transformed TTC, we used a linear mixed model (LMM) accounting for repeated observations of individual differences by including individuals as a random factor. Main effects and two-way interactions were tested separately by stepwise backward term removal based on the Akaike information criterion (AIC) and likelihood ratio test to compare models and determine significant terms (Bolker et al., 2009). To estimate degrees of freedom the Satterthwaite approximation (Satterthwaite, 1946) was used. Residuals were tested for normality using the Shapiro test and for homogeneity visually. Post hoc analyses of significant main effects and interactions were performed by multiple *t* tests with Bonferroni correction. The reversal index is considered to measure the difficulty of the reversal (Rajalakshmi & Jeeves, 1965; Warren, 1967) and is defined as $\frac{1\;+\text{ TTC required in the reversal}}{1\;+\text{ TTC required for the acquisition}}$. This index was calculated at the individual level and compared between conditions using a Wilcoxon signed-rank test.

In a post hoc linear mixed model we introduced TTC required for the initial acquisition (TTCac) as an additional term. This model tested for the effect of TTCac on the difference between trials required in acquisition and trials required in the reversal (ΔTTC) by single-term deletion and likelihood ratio testing. TTCac and ΔTTC were transformed in the same way as TTC to meet assumptions of linearity.

Statistical analyses were performed using R 3.0.2 (R Core Team, 2013) software. For modelling, we used the ‘lme4’ package (Bates, Maechler, Bolker, & Walker, 2013) and for visual presentation of data ‘ggplot2’ (Wickham, 2009).

## Results

**Figure 2:**
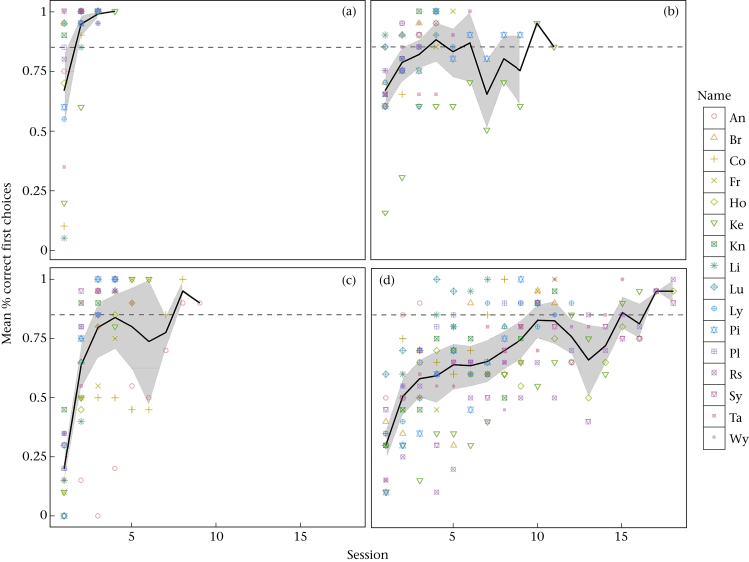
Learning curves in different phases and conditions: (a) acquisition with solid objects; (b) acquisition on the touchscreen; (c) reversal with solid objects; (d) reversal on the touchscreen. Black lines represent the mean performance of all individuals, symbols represent individual performance, grey areas denote confidence limits of the population means; dashed lines indicate the learning criterion.

Our model revealed significant main effects of phase and condition, as well as significant interactions of stimulus set used with condition, group with condition and condition by phase (see Table 2 for detailed results of all factors and interactions). Overall, individuals required more trials to reach the criterion in the reversal (mean = 141, SE = 18.22) than in the acquisition (mean = 45.72, SE = 11.44; LMM: *b* = 1.513, *t*_44.99_ = 8.097, *P* < 0.01). They also required more trials to learn and reverse the discrimination of images (mean = 143.84, SE = 19.73) than of solid objects (mean = 42.88, SE = 10.50; LMM: *b* = 1.522, *t*_44.99_ = 8.146, *P* < 0.01; compare shape and length of learning curves in Fig. 2a, b, c, d).

**Figure 3:**
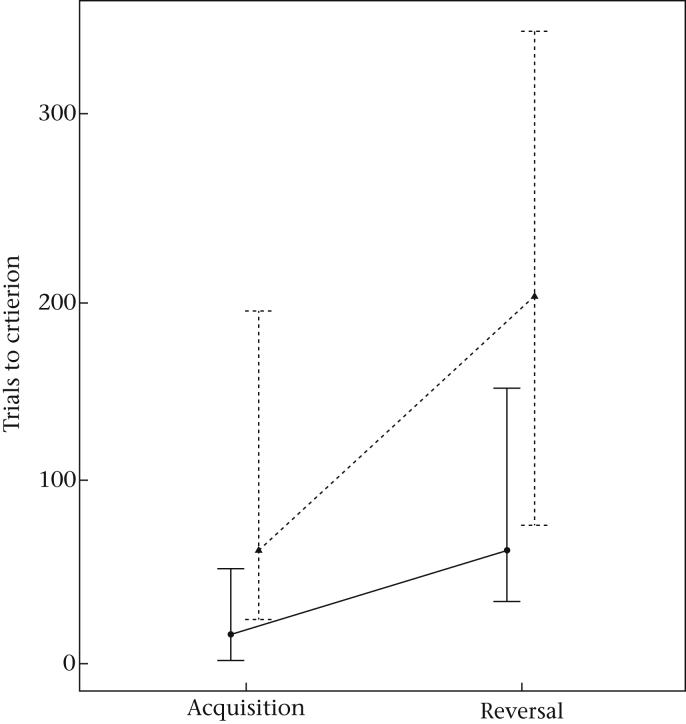
Median trials to criterion with 95% confidence intervals for the interaction of phase with condition. Performance on the touchscreen is represented by dashed lines and triangles; solid objects are represented by full lines and circles.

**Figure 4:**
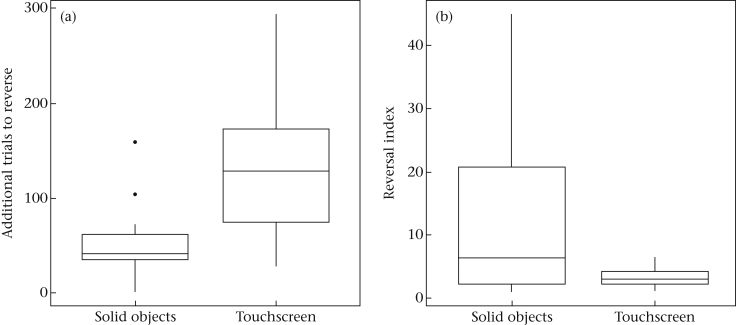
Box plots of (a) number of trials required to reach criterion in the reversal phase subtracted by the number of trials required in the initial acquisition phase (ΔTTC) for each condition and (b) reversal index $\left(\frac{1\;+\;TTC\;required\;in\;the\;reversal}{1\;+\;TTC\;required\;for\;the\;acquisition}\right)$ for both conditions. Thick horizontal lines indicate median values, boxes span from the first to the third quartiles and the whiskers represent 95% confidence intervals; outliers are denoted by dots.

Post hoc tests of the interaction of phase and condition (LMM: *b* = −0.796, *t*_42.99_ = −2.320, *P* = 0.025) revealed significant differences between acquisition and reversal with solid objects (*P* < 0.01), acquisition and reversal on the touchscreen (*P* < 0.01) and between the conditions in both the acquisition (*P* < 0.01) and the reversal phase (*P* < 0.01; see Fig. 3). Individuals required more trials to reach criterion in the reversal on the touchscreen than in the reversal with solid objects yielding a significant difference between conditions (*t*_15_ = −2.83, *P* = 0.013, *r* = 0.59; Fig. 4a). However, performance with solid objects produced significantly higher reversal indexes than on the touchscreen (*P* = 0.015, *r* = −0.42; Fig. 4b).

To investigate the effect of extended training of the initial discrimination on the reversal performance, we included the trials required to reach criterion in the acquisition of discrimination as a factor in a post hoc model. This model revealed no significant effect of TTC in the acquisition on trials required for the reversal (Χ^2^_1_ = 2.53, *P* = 0.112), while the effect of condition remained significant (Χ^2^_1_ = 12.27, *P* < 0.01).

We also investigated possible sequence effects of the conditions. The interaction of group with condition (LMM: *b* = 0.820, *t*_42.99_ = 2.236, *P* = 0.03) revealed in post hoc testing a significant difference in TTC between conditions when the touchscreen was the first condition (mean_solid objects_ = 39.55, SE_solid objects_ = 9.77; mean_touchscreen_ = 135.4, SE_touchscreen_ = 20.94; *P* < 0.01), but only a modest tendency to differ if individuals started in the solid object condition (mean_solid objects_ = 48.42, SE_solid objects_ = 7.75; mean_touchscreen_ = 157.92, SE_touchscreen_ = 32.22; *P* = 0.086). For the performance in both the solid object (*P* = 0.37) and the touchscreen (*P* = 1.00) conditions, it did not matter which condition a subject started with.

Post hoc analysis of the interaction of stimulus set with condition (LMM: *b* = 0.919, *t*_13_ = 2.169, *P* = 0.05) showed a significant difference of TTC between conditions for the yellow and red stimulus set (mean_solid objects_ = 34.82, SE_solid objects_ = 7.13; mean_touchscreen_ = 137.63, SE_touchscreen_ = 20.91; *P* < 0.01) as well as the blue and orange stimulus set (mean_solid objects_ = 50.94, SE_solid objects_ = 11.28; mean_touchscreen_ = 150.06, SE_touchscreen_ = 28.96; *P* < 0.01). Performance between these sets did not differ either in the solid object (*P* = 0.31) or in the touchscreen (*P* = 1.00) condition.

## Discussion

Comparing the discrimination and reversal performance of kea either manipulating three-dimensional objects or choosing two-dimensional representations thereof presented on a touchscreen, we found contradictory results regarding absolute or relative number of trials required to reach the learning criterion. It seems that the first, more natural method is easier for discriminative purposes resulting in few trials required for acquisition. We attribute this difference in difficulty to additional stimulus qualities such as depth cues and the haptic dimension, allowing for overall better discrimination of solid objects. However, this advantage in discriminative abilities seems to affect the reversal negatively in relation to the acquisition of the task. A less accurate discrimination of the image stimuli benefits the individuals in the reversal allowing for a relatively faster reversal in comparison to the trials required for the acquisition, hence making the reversal less difficult in this condition. This is also reflected by the greater reversal index with solid objects.

However, the fact that the absolute increase in trials required from acquisition to reversal was larger on the touchscreen than with solid objects allows us to argue for a mechanism acting on the inhibition of responses towards the former rewarded stimulus rather than on the formation of a new association as has been previously suggested for position and colour reversals (Macphail, 1971, 1976). Interestingly, it seems that this effect is more pronounced on the touchscreen. More sessions required in the acquisition may be considered equivalent to greater exposure to the task and therefore creating a stronger association with the positive stimulus. Therefore, inhibition of responses to the former rewarded stimulus in the reversal might require more time (Mackintosh, 1963). Since we could show that the length of the acquisition, measured in terms of trials to criterion (TTC), did not affect the number of additional trials required in the reversal, we believe this hypothesis can be ruled out.

The spatial relationship between stimulus, response and reward has been shown to be of major importance in discrimination learning (Miller & Murphy, 1964). While in the solid object condition the reward was always placed underneath the cups, and therefore in close proximity of the stimulus, in the touchscreen condition it dropped out from the food dispenser at an equal distance from the positive and the negative pictures on the screen. Miller and Murphy (1964) showed significantly faster acquisition when the reward was connected to the stimuli compared to a condition in which reward and stimuli were separated. Here we argue that the spatial relation between stimulus and reward not only affects the acquisition of a discrimination task, but also the inhibition of the previously learned responses. In this respect the solid object condition benefits the reversal by additional immediate feedback, which may be used for inferring the reward location, thus allowing the birds to avoid checking the unrewarded stimulus twice. The effect of the spatial relation between cue and reward (a direct one in solid objects and an ambiguous visuospatial feedback on the touchscreen), promoting repeated responses towards the unrewarded stimulus, is also reflected in the greater number of errors on the touchscreen. We therefore argue that the touchscreen approach promotes preservative responses, whereas inhibition and association formation are aided by inferences based on more proximate visuospatial feedback within solid objects.

While pictures presented on the touchscreen can only be discriminated visually in two dimensions, solid objects can be discriminated visually in three, providing depth cues and overall more informational content (see Aust & Huber, 2006; Fagot, 2000; Watanabe, 2000, 2006). In this respect, Stephan et al. (2014) showed that when pigeons were presented with holograms of objects, which provided them with depth cues, the subjects were equally able to discriminate objects in both presentational modes and exhibited perceived equivalence of both stimulus types.

Furthermore, it is not clear how the kea exactly perceive the colours on the touchscreen. In this respect, the interaction between stimulus set and condition supports the argument for different informational content of the stimuli. This can be explained by a preference for the blue stimulus (used in the acquisition) on the touchscreen. Such a preference may be balanced out by further stimulus qualities or alternative colour perception in solid objects.

Objects can also be discriminated on the basis of olfactory cues (Uwano, Nishijo, Ono, & Tamura, 1995) and kea, indeed, have a well-developed sense of smell (Gsell, Hagelin, & Brunton, 2012; Steiger, Fidler, Valcu, & Kempenaers, 2008). However, their poor performance in the first trials of the solid objects reversal makes the use of olfactory cues for the discriminations unlikely.

The kea participating in this study were experienced with tasks involving solid objects, as they had been tested extensively on their sophisticated physical intelligence (for a review see Huber & Gajdon, 2006). We also note here that birds in general have much greater experience with food cues provided by solid objects becoming invalid than our captive subjects had, solely due to their test experience. Thus it is implicit that any long-lived subject has ample experience with solid objects and is naïve to touchscreen procedures rather than being equally experienced with both kinds of stimuli. Nevertheless, previous experience with a reversal learning task using solid objects (Gajdon et al., 2011) did not influence their performance. Similarly, previous experience in a discrimination task on the touchscreen (O'Hara et al., 2012) had no effect on the performance in the current reversal learning task. However, based on the interaction between group and condition, we suggest that the direct previous experience did have an improving effect on the performance in the subsequent condition. A transfer of knowledge about the task may explain the observed improvement in performance, from one condition to the other, by the formation of learning sets (Harlow, 1949; Zimmermann & Hochberg, 1971). The most likely explanations for the overall effect of condition are additional sensory (especially haptic) information, more experience with objects than with pictures and a less obvious spatial relationship between pictures and reward than between objects and reward.

The finding that overall reversals take longer than the acquisition is in line with the literature and different brain lesion studies have concluded that poor reversal learning following Wulst lesions is caused by impaired inhibition (Dias et al., 1996; Lai et al., 1995; Macphail, 1971, 1976). Earlier, Macphail (1975) suggested deficits after lesions to be due to an inability to shift attention. Such difficulties to switch to former unrewarded stimuli, rather than inability to overcome inhibition, have also been suggested by Powers (1989). Bonté, Kemp, and Fagot (2014) have suggested that inhibitory control and cognitive flexibility might involve different cognitive processes, since studies on age-dependent advancements of inhibitory control (Fagot, Bonté, & Hopkins, 2013) contradict age-related decline in cognitive flexibility (Bonté, Flemming, & Fagot, 2011; Bonté et al., 2014). Furthermore, since the age-dependent patterns in a reversal learning task (Bonté et al., 2014) resembled those in the Wisconsin Card Sorting Task (Bonté et al., 2011), Bonté et al. (2014) concluded that reversal learning rather measures cognitive flexibility. Watanabe (2012) also proposed that reversal learning performance could be considered a measure of cognitive flexibility, a strategy of animals facing an unstable environment. Bond et al. (2007, p. 373) distinguished between three connotations of flexibility and suggested ‘flexibility of behaviour pattern’ supposedly ‘subsumes all the cognitive abilities required for serial reversal learning’. Based on their results Bond et al. (2007) concluded that behavioural flexibility is associated with social complexity rather than ecological or spatial complexity. However, Tebbich et al. (2010) did find influences of a species' feeding ecology on number of errors in the reversal. Woodpecker finches, *Cactospiza pallida*, committed significantly more preservative errors than small tree finches, *Camarhynchus parvulus*, which the authors attributed to the woodpecker finches' foraging style. The urge to occasionally, but consistently, recheck a former rewarded stimulus fits the ecological requisites of an extractive forager, such as the kea.

While impaired discriminative abilities on the touchscreen account for overall greater number of errors and more trials required to reach the criterion, we suggest that persistency is responsible for the increased difficulty of the reversal in the more natural condition considering the reversal index. A comparison with the results from Lissek, Diekamp, and Güntürkün (2002), Bond et al. (2007) and Tebbich et al. (2010) indicate that this effect might be generally more pronounced in species that are considered extractive foragers. These species often rely on persistency in order to access food, which may overrule and impair inhibition in certain contexts. However, in this species comparison the kea rank among the fastest (in the acquisition of solid objects) and slowest learners (in the reversal on the touchscreen). Generally the kea show the greatest reversal index, which we attribute to their persistency. The fact that they are at the same time still the fastest to reverse in absolute terms, at least with solid objects, we consider an evolutionary result of their complex and rapidly changing environment.

This study has been a first attempt to investigate the validity of direct comparisons of the same paradigm employing the standard versions of two approaches. While we can exclude the two methods being directly comparable, the cause of these differences remains suggestive and subject to future research. However, we argue that such direct comparisons (as they are frequently being made in the literature) have to be treated with great caution. The touchscreen seems to be the more difficult condition for discrimination learning due to limited stimulus qualities and spatial relation between stimulus and reward. While such impaired discriminative abilities on the touchscreen promote shifting of attention, thus allowing for faster relative reversal, direct visuospatial feedback increases inhibitory abilities and hence faster absolute reversal performance with solid objects. In this respect it is important to keep in mind that kea are not pigeons and further research is required to understand exactly how much such manipulative species rely on tactile feedback. To conclude, we argue that the evaluation of flexibility employing the touchscreen approach does not necessarily reflect individuals' capacities employed in the ‘real world’. This might, in particular, be the case for species relying on additional sensory modalities, supporting visual perception. In this respect our results demonstrate the importance of considering a species' ecology when devising and interpreting such cognitive tasks.

## Figures and Tables

**Table 1:** Individuals participating in the experiment with their full names and abbreviations in parentheses

Name	Hatched	Sex	SO reversal	TS experience	Starting condition (group)	Starting set (stimulus set)
Anu (An)	2007	♂	Yes	Training	Touchscreen	Set 1
Bruce (Br)	2002	♂	Yes	Discrimination	Touchscreen	Set 2
Coco (Co)	2007	♀	Yes	Training	Touchscreen	Set 1
Frowin (Fr)	2004	♂	Yes	Training	Touchscreen	Set 2
Hope (Ho)	2007	♀	Yes	Training	Solid objects	Set 1
Kermit (Ke)	2004	♂	Yes	Discrimination	Touchscreen	Set 1
Knut (Kn)	2000	♂	Yes	Training	Solid objects	Set 1
Lilly (Ly)	2007	♀	No	Training	Solid objects	Set 1
Linus (Li)	2004	♂	Yes	Training	Solid objects	Set 2
Luke (Lu)	2003	♂	Yes	Discrimination	Touchscreen	Set 2
Mismo (Mi)	1999	♂	Yes	Training	Solid objects	Set 1
Pick (Pi)	2004	♂	Yes	Discrimination	Touchscreen	Set 1
Plume (Pl)	2007	♀	No	Discrimination	Touchscreen	Set 1
Roku (Ro)	2008	♂	No	Training	Solid objects	Set 2
Rosa (Rs)	2001	♀	No	Discrimination	Solid objects	Set 1
Rudy (Ry)	2007	♀	Yes	Training	Solid objects	Set 2
Sunny (Sy)	2007	♀	Yes	Training	Touchscreen	Set 2
Tammy (Ta)	2007	♂	Yes	Training	Solid objects	Set 2
Willy (Wy)	2007	♀	Yes	Discrimination	Touchscreen	Set 2
Zappel (Za)	2004	♂	Yes	No	Solid objects	Set 2

SO reversal indicates individual participation in a prior tool use reversal task with solid objects (Gajdon et al., 2011). Prior touchscreen (TS) experience is coded ‘training’ if they only had habituation training and ‘discrimination’ if they had also participated in the pilot study (see O'Hara et al., 2012). The starting condition shows whether the individual started in the touchscreen or solid object condition, and starting set means which stimulus set (1 or 2) was used first.

**Table 2:** Test statistics of single-term deletions showing the effect of each factor or interaction term on the model's fit, compared to the full model by likelihood ratio testing

		*df*	AIC	χ^2^	*P*(χ^2^)	
Main effects	<None>		162.11			
	Condition	1	202.80	42.691	<0.001	***
	Phase	1	202.44	42.329	<0.001	***
	Group	1	162.11	3.822	0.051	†
	Stimulus set	1	160.29	2.458	0.117	
	Age	1	159.83	0.056	0.813	
	Sex	1	161.77	0.005	0.943	
	Solid object – pre-experience	1	163.77	0.248	0.618	
	Touchscreen – pre-experience	1	165.52	0.081	0.777	
Two-way interactions	Stimulus set×condition	1	161.06	7.276	0.006	**
	Phase×condition	1	159.45	5.667	0.017	*
	Group×condition	1	159.06	5.284	0.022	*
	Group×phase	1	155.78	2.483	0.115	
	Sex×phase	1	155.30	0.701	0.402	
	Age×condition	1	156.60	0.507	0.476	
	Sex×condition	1	158.09	0.641	0.423	
	Age×phase	1	159.45	0.049	0.824	
	Stimulus set×phase	1	161.40	0.008	0.929	

AIC gives the model fit (employing the Akaike information criterion) for any single term excluded. †*P* < 0.1; *P < 0.05; **P < 0.01; ***P < 0.001.
